# An *in vivo* humanized model to study homing and sequestration of *Plasmodium falciparum* transmission stages in the bone marrow

**DOI:** 10.3389/fcimb.2023.1161669

**Published:** 2023-04-19

**Authors:** Samantha Donsante, Giulia Siciliano, Mariagrazia Ciardo, Biagio Palmisano, Valeria Messina, Valeria de Turris, Giorgia Farinacci, Marta Serafini, Francesco Silvestrini, Alessandro Corsi, Mara Riminucci, Pietro Alano

**Affiliations:** ^1^ Department of Molecular Medicine, Sapienza University of Rome, Rome, Italy; ^2^ Dipartimento Malattie Infettive, Istituto Superiore di Sanità, Rome, Italy; ^3^ Center for Life Nano- and Neuro-Science Istituto Italiano di Tecnologia, Rome, Italy; ^4^ Centro Ricerca M. Tettamanti, Department of Pediatrics, University of Milano-Bicocca, Monza, Italy

**Keywords:** malaria, *Plasmodium falciparum*, gametocytes, bone marrow, ectopic ossicles, skeletal stem cells, bone marrow adipocytes, organoids

## Abstract

**Introduction:**

Recent evidence suggests that the bone marrow (BM) plays a key role in the diffusion of *P. falciparum* malaria by providing a “niche” for the maturation of the parasite gametocytes, responsible for human-to-mosquito transmission. Suitable humanized *in vivo* models to study the mechanisms of the interplay between the parasite and the human BM components are still missing.

**Methods:**

We report a novel experimental system based on the infusion of immature *P. falciparum* gametocytes into immunocompromised mice carrying chimeric ectopic ossicles whose stromal and bone compartments derive from human osteoprogenitor cells.

**Results:**

We demonstrate that immature gametocytes home within minutes to the ossicles and reach the extravascular regions, where they are retained in contact with different human BM stromal cell types.

**Discussion:**

Our model represents a powerful tool to study BM function and the interplay essential for parasite transmission in *P. falciparum* malaria and can be extended to study other infections in which the human BM plays a role.

## Introduction

The human bone marrow (BM) organ is comprised of hematopoietic tissue and a three-dimensional network of non-hematopoietic cell types collectively referred to as the BM stroma (Tavassoli and Yoffey, 1984). The ultimate function of the BM is to preserve hematopoietic stem cells (hematopoietic niche) and to provide cues for the differentiation of committed progenitors into mature blood cells (hematopoietic microenvironment) (Bianco et al., 2011). However, increasing evidence suggests that due to its unique cellular and molecular milieu and multifunctional stroma, which remarkably includes post-natal skeletal stem and progenitor cells, the BM is also a critical player in a variety of physiological processes and in many systemic pathological conditions, especially neoplastic (Granata et al., 2022) and infectious diseases (Perng, 2012; Pathak and Das, 2021). Indeed, the selective engraftment of some types of cancer cells in the BM as well as its engulfment with microorganisms in some chronic systemic infections are long standing notions in human pathology. However, while the frequent colonization by cancer cells has prompted intensive investigation to better understand the pathogenetic role of the BM in human oncology, the contribution of this organ to infectious diseases is still far less clear.

An unexplored aspect of BM biology currently receiving increasing attention is its role in hosting the transmission stages of Plasmodium (Venugopal et al., 2020), the protozoan parasite causing the devastating malaria disease that counted only in 2021 over 240M cases and 630,000 victims, mainly in African children (World malaria report 2021, n.d.). In the most severe form of the disease, due to *Plasmodium falciparum*, the asexual stage parasites proliferate within red blood cells and adhere to the microvascular endothelium of virtually all internal organs, causing malaria pathology (Moxon et al., 2020). At every multiplication cycle, a fraction of parasites stops proliferating and differentiates as gametocytes, the parasite sexual stages transmissible to Anopheles mosquitoes. In *P. falciparum*, the 10-day-long process of gametocyte maturation is conventionally divided in five morphological stages (I-V), of which only the mature stage V gametocytes are found in circulation in peripheral blood (Hawking et al., 1971). Early malariology observations had revealed that all immature stages of gametocytogenesis are readily found in the BM (Bastianelli and Bignami, 1899) but only recently the analysis of autoptic specimens (Joice et al., 2014), BM aspirates (Aguilar et al., 2014) and one clinical case (Farfour et al., 2012) confirmed that the *P. falciparum* gametocytes preferentially accumulate in this organ and observed that, intriguingly, the immature gametocytes are not confined inside the microvasculature but are also found within the BM extravascular compartment, in the BM stroma (Farfour et al., 2012; Joice et al., 2014).

The cellular and molecular mechanism(s) governing gametocyte homing to and maturation in the BM are unknown. Immature (stage I-IV) gametocytes fail to adhere to human endothelial cells derived from several organs, including BM, in conventional 2D cell binding assays (Silvestrini et al., 2012; Tibúrcio et al., 2013), whereas stage II-III gametocytes can establish physical and regulatory interactions with human BM derived skeletal stem/progenitor cells in a tridimensional co-culture system (Messina et al., 2018). This suggests the need to investigate this elusive host parasite interplay in systems recapitulating at best the complexity of the human BM anatomy and physiology.

Based on recent advances in bioengineering, attempts have been made to generate BM-on-a-chip models. However, difficulties in reproducing fundamental anatomical features of the BM compartment, such as its vascular architecture, and the variability in cell seeding caused by the small volume of the chamber (Glaser et al., 2022) are still major drawbacks of the chip-based approach. In contrast, the ectopic human ossicle represents a well-established experimental method that exploits the dual nature, as osteoprogenitor cells and tissue organizers, of human BM-derived skeletal stem cells to reproduce the structure and function of the human BM in immunocompromised mice (Sacchetti et al., 2007; Serafini et al., 2014; Pievani et al., 2017; Rostovskaya et al., 2018; Donsante et al., 2021; Pievani et al., 2021). Different protocols are currently in use based on *in vivo* transplantation of either cell-scaffold constructs containing undifferentiated skeletal stem cells (Sacchetti et al., 2007; Rostovskaya et al., 2018) or skeletal stem cell-derived cartilaginous pellets (Serafini et al., 2014; Pievani et al., 2017; Pievani et al., 2021). Although both procedures culminate in the establishment of a chimeric ossicle with donor-derived BM stroma, the scaffold-free method based on cartilage transplantation offers the opportunity to investigate cell functions and interactions within the ossicle in the absence of exogenous materials. Cartilaginous pellets generated *in vitro* completely derive from human progenitor cells and after *in vivo* transplantation they are converted into a chimeric ossicle with a marrow cavity, containing stromal cells and adipocytes of human origin and blood vessels and hematopoiesis derived from the mouse host (Serafini et al., 2014). Therefore, with the aim to establish an *in vivo* mouse model of the *P. falciparum* gametocyte-human BM interplay, we used this model to investigate the homing to and distribution of immature gametocytes within the humanized ossicle.

## Materials and methods

### Parasite culture and gametocyte production

The *P. falciparum* 3D7 (Walliker et al., 1987) line and the Pf2004/164-tdTom line (Brancucci et al., 2015) were cultured in human 0+ erythrocytes, kindly provided by Prof. A. Angeloni, Immunohematology and Transfusion Medicine, Policlinico Umberto I, Sapienza University of Rome, at 5% hematocrit under 5% CO_2_, 2% O_2_, 93% N_2_ (Trager and Jensen, 1976). Cultures were grown in RPMI 1640 medium (Gibco, Thermo Fisher Scientific, Waltham, MA, USA) supplemented with 25 mM Hepes, 50 µg/ml hypoxanthine, 0.25 mM NaHCO_3_, 50 µg/ml gentamicin sulfate and 10% pooled heat-inactivated 0+ human serum (IBB, Memphis, TN, USA).

Cultures were induced to produce gametocytes *via* parasite overgrowth; after appearance of the oat shaped stage I gametocytes a 72h, treatment with 50 mM N-acetyl-glucosamine (NAG) was applied to eliminate residual asexual parasites (Gupta et al., 1985). Stage II-III gametocytes were purified from uninfected erythrocytes on MACS Separation Columns CS (Miltenyi Biotec Bologna, Italy) as previously reported (Ribaut et al., 2008).

### Human skeletal stem/progenitor cell isolation and culture

Cultures of non-hematopoietic, adherent cells (bone marrow stromal cells, BMSCs or mesenchymal stromal cells, MSCs) were established from iliac crest BM aspirates from healthy donors (n=2) and used under a protocol approved by the Ethics Committee of San Gerardo Hospital-Monza after written informed consent was obtained.

Mononuclear cells were isolated using a Ficoll-Paque™ Plus (GE Healthcare, Little Chalfont, UK) density gradient separation and seeded at clonal density (2x10^5^ cells/cm^2^) in Dulbecco’s modified Eagle’s medium (DMEM)-LG (Thermo Fisher Scientific), 10% fetal bovine serum (FBS) (Thermo Fisher Scientific), 1% Penicillin-Streptomycin (Thermo Fisher Scientific), and 1% L-glutamine (Thermo Fisher Scientific). The next day, medium was replaced to remove non-adherent (hematopoietic) cells. After one week, BMSCs grew as discrete colonies, among which skeletal stem/progenitor cells are included. The first passage was performed on days 12-16 when colonies began to merge. Cultures were incubated at 37°C in a 5% CO_2_ atmosphere (Kuznetsov et al., 1997) and medium was changed 2 times per week.

### Generation of cartilaginous pellets

Pellet cultures were established as previously described (Serafini et al., 2014; Pievani et al., 2017). Briefly, multiclonal BMSC populations at the 2nd or 3rd passage were grown for 3 weeks as unmineralized pellets in 15 ml polypropylene conical tubes at a density of 3 × 10^5^ cells/tube in Chondrogenic Differentiation Medium [CDM; DMEM-High glucose (Thermo Fisher Scientific) supplemented with ITS™ premix (BD Biosciences, San Jose’, CA, USA), 1 mM pyruvate (Sigma-Aldrich, St. Louis, MO, USA), 50 μg/ml 2-phosphate–ascorbic acid (Sigma-Aldrich), 100 nM dexamethasone (Sigma-Aldrich), and 10 ng/ml TGFβ1 (Sigma-Aldrich)]. Chondrogenic differentiation was evaluated by histological analysis of formalin-fixed, paraffin-embedded pellets stained with Toluidine blue.

### *In vivo* transplantation of cartilaginous pellets

Ectopic ossicles with a human BM stroma were generated by transplantation of cartilaginous pellets into immunocompromised mice (SCID/beige, CB-17/IcrHsd-Prkdc^scid^Lyst^bg^, Charles River, Wilmington, MA, USA)

All animal procedures were authorized by the Ministry of health (n. 574/2017-PR, 17-07-2017) after approval of the ISS National Centre for Animal Experimentation and Welfare (BENA).

Pellet transplantation was performed as previously described (Serafini et al., 2014; Pievani et al., 2017). Briefly, up to 4 chondroid pellets were transplanted subcutaneously into 8- to 15-week old female SCID/beige mice, previous anesthetized with an intramuscular injection of Zoletil ^®^ 20 (Virbac, 5 mL/g of body weight) and harvested after 8 weeks.

### *P. falciparum* gametocyte injection in SCID/beige mice

In order to measure the clearance from circulation, 1x10^7^ stage II-III purified gametocytes obtained as described above from the *P. falciparum* line 3D7 were stained with the PKH26 red fluorescent dye (Sigma-Aldrich), resuspended in 200 µl of PBS and intravenously injected in SCID/beige mice (n=2). About 20 μl of peripheral blood were collected from each mouse 2, 10, 30 and 120 minutes after injection, 10 μl of which were resuspended in 180 μl of PBS to be analyzed by Flow Cytometry (FACSCalibur™-BD Biosciences) and 10 μl were smeared on a slide for Giemsa staining and microscopic analysis.

To study the sequestration of gametocytes in the ossicles and in mice mouse organs, 0.3-1 x 10^7^ stage II-III gametocytes (Pf2004/164-tdTom line) were resuspended in 200 μl of PBS and intravenously inoculated in BM humanized mice (n = 8). Mice were sacrificed at 10 minutes (n=3), 1 hour (n=3) and 24 hours (n=2) post injection.

In order to assess whether non-vital gametocytes were able to colonize the ossicle, stage II-III gametocytes were treated with 2 µM methylene blue (MB) (Siciliano et al., 2017) before injection and mice were sacrificed 10 minutes (n=2) and 1hr (n=2) later.

### Histology

Chimeric ossicles, mouse bone samples and organs were harvested immediately after mouse sacrifice and fixed overnight in 4% formaldehyde in PBS pH 7.4. Then ossicles and bone samples were decalcified in 10% EDTA for 2 weeks. For embedding, all samples were placed in 20% sucrose and 2% Polyvinylpyrrolidone (PVP) in PBS for 48 hr and embedded in an 8% porcine gelatin solution containing 20% sucrose and 2% PVP as previously reported (Kusumbe et al., 2015; Palmisano et al., 2022). Five to 10 μm thick sections were cut with a cryostat and allowed to air-dry. For standard histology, sections were stained with hematoxylin and eosin (H&E) and imaged with a Zeiss Axiophot microscope (Carl Zeiss). For confocal microscopy, sections were stained with TO-PRO-3 (T3605, Thermo Fisher Scientific) for visualization of nuclei and analyzed with a Leica Confocal Microscope (Wetzlar, Germany).

### Immunostaining

Ten-μm-thick sections were air-dried, hydrated with PBS and exposed to 5% goat serum in PBS for 30 minutes at RT. For immunostaining of gametocytes, the sections were incubated with a mouse monoclonal anti-Pfs16 antibody (Bruce et al., 1994) (32F717-SF, kindly provided by Dr. R. Sauerwein, Radboud University Medical Centre, Nijmegen, The Netherlands), at a dilution of 1:500 in PBS, O/N at 4°C. For immunostaining of cells within the ossicles the following antisera were used: rabbit polyclonal anti-mouse and human Fatty Acid Binding Protein (FABP4, ab13979, Abcam Cambridge, UK), anti-human CD146 (ab75769, Abcam), anti-human Alkaline Phosphatase (ALP, 11187-1-AP, ProteinTech Manchester, UK) all applied at 1:100 in PBS, O/N at +4°C. Following washing with PBS, samples were incubated with Alexa Fluor 647-conjugated goat anti-mouse (A-21240, Thermo Fisher Scientific) or Alexa Fluor 488-conjugated goat anti-rabbit (A-11008, Thermo Fisher Scientific) at dilution of 1:200 in PBS for 1 hr at RT. Nuclei were counterstained with TO-PRO-3.

### *P. falciparum* gametocyte quantification

The number of stage II-III TdTomato gametocytes per ossicle marrow area and the number of stage II-III TdTomato gametocytes in the intra- and extra-vascular ossicle compartment was evaluated on confocal microscopy images taken at 40x magnification, by two independent operators. All measures were performed by using ImageJ software.

### Three-dimensional confocal microscopy analysis

For three-dimensional confocal microscopy analysis, 50-µm-thick sections were immunostained with anti-FABP4 antibody and images were acquired at 1024x1024px by an Olympus iX83 FluoView1200 laser scanning confocal microscope using an UPLSAPO60x oil NA1,35 (Olympus), 473 nm, 559 nm and 635 nm lasers and a z-step of 200 nm. The image stacks were visualized as three-dimensional (3D) reconstruction performed with Imaris v8.1.2 software (Bitplane). For 3D volume rendering, the “Surfaces” tool was used to generate 3D structures corresponding to *P. falciparum* gametocytes (red) and vessel (green); to highlight the 3D structures, DIC transmitted light and nuclei staining (purple) was shown using “Ortho slicer” feature.

## Results

### Stage II-III gametocytes are rapidly cleared from mouse peripheral blood

**Figure 1:**
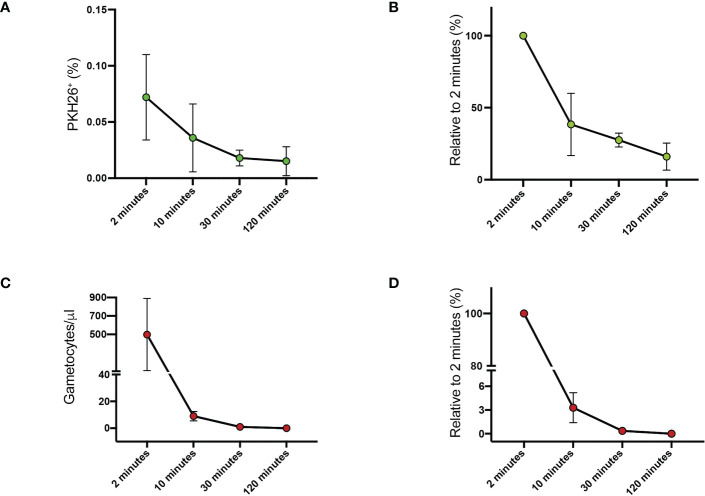
Clearance of PHK26+ P. falciparum stage II-III gametocytes from mouse peripheral blood at 2, 10, 30 and 120 minutes **(A)** Average of the flow cytometry counts from the two mice. **(B)** Flow cytometry counts of stage II-III gametocytes expressed as percentage relative to 2-minute value. **(C)** Stage II-III gametocytes per μl counted on Giemsa-stained blood smears. **(D)** Stage II-III gametocytes counted on Giemsa-stained blood smears expressed as percentage relative to 2-minute value. All data in **(A–D**) are presented as the mean ± SEM.

We first assessed the kinetics of clearance of *P. falciparum* gametocytes from mouse peripheral blood. Purified gametocytes of the *P. falciparum* reference line 3D7 (Walliker et al., 1987) at stage II-III of maturation were stained with the PKH26 fluorescent dye. A suspension of 10^7^ gametocytes was injected in the tail vein of SCID/beige mice and 20 µl of peripheral blood were collected 2, 10, 30 and 120 minutes later. Blood samples were examined by flow cytometry and by transmitted light microscopy on Giemsa-stained smears. Both analyses showed that gametocytes were rapidly cleared from circulation. At 2 minutes, only 0.072% ± 0.038 of injected gametocytes were detected by flow cytometry in circulating blood (**Figure 1A**, **Supplementary Figure S1**). Thereafter, a progressive reduction of 62%, 73% and 84% relative to 2 minutes was observed at 10, 30 and 120 minutes respectively (**Figure 1B**, **Supplementary Figure S1**). Similarly, 497.75 ± 391.45 gametocytes per microliter of blood were detected by Giemsa stain at 2 minutes (**Figure 1C**), but only the 3.28% of this value was still detectable at 10 minutes (**Figure 1D**).

### *P. falciparum* immature gametocytes localize in the ectopic ossicle

To explore the ability of *P. falciparum* gametocytes to localize in chimeric ossicles generated in immunocompromised mice, we used gametocytes produced by the *P. falciparum* transgenic line Pf2004/p164-tdTom (Brancucci et al., 2015) in which the tdTomato reporter gene is chromosomally integrated in the genome and is expressed from stage I-II of gametocytogenesis.

**Figure 2:**
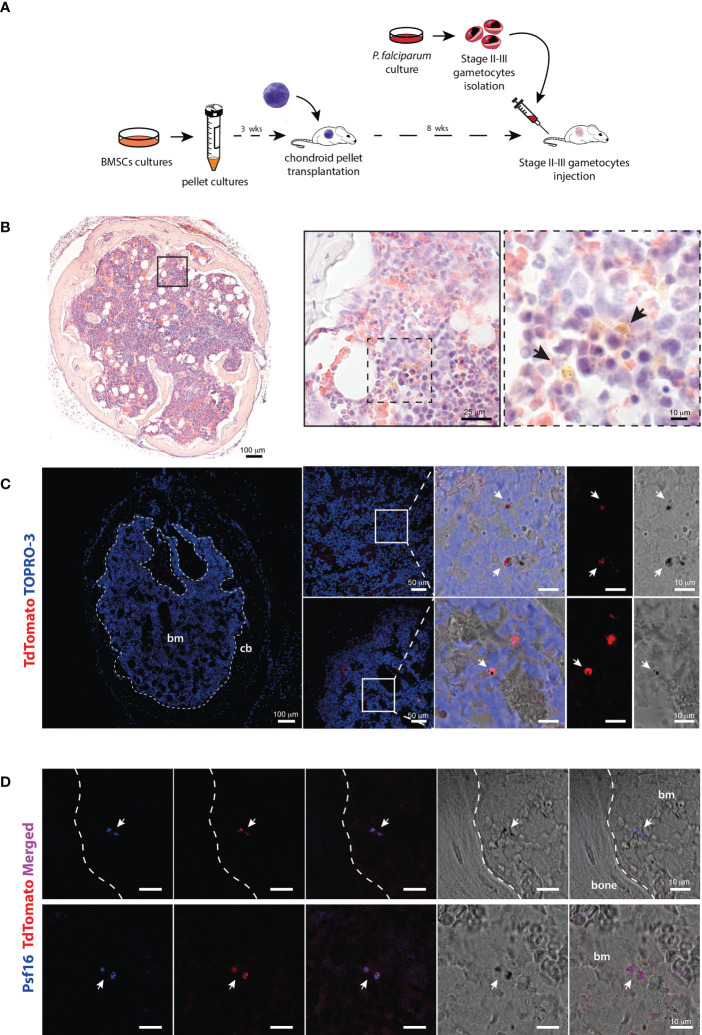
Identification of *P. falciparum* stage II-III gametocytes in the chimeric ossicle **(A)** Schematic representation of the experimental procedure for the generation of BM humanized mouse model and injection of Stage II-III gametocytes. **(B)** H&E-stained ossicle section showing the presence of black-brown pigmented structures consistent with hemozoin in the marrow (arrows). **(C)** Confocal fluorescent microscopy images showing the presence of TdTomato positive and pigmented structures within the bone marrow ossicle (white arrows). **(D)** Immunostaining for the gametocyte specific protein Psf16 confirming the presence of stage II-III gametocytes. Fluorescent images show overlapping signal of antibody anti-Psf16 (blue), TdTomato (red) and black pigments. All the ossicles were harvested 10 minutes after gametocytes injection. bm, bone marrow; cb, cortical bone.

Purified stage II-III gametocytes were injected in the tail vein of immunocompromised mice 8 weeks after transplantation of cartilaginous pellets, when cartilage was completely replaced by bone and marrow cavities (**Figure 2A**). Since the time of clearance from the blood suggested a rapid peripheral distribution of gametocytes, the sacrifice was performed 10 minutes after the injection.

Multiple mouse organs including liver, spleen, lungs, kidneys, brain and bone marrow were harvested, embedded in gelatin and examined by fluorescent confocal microscopy. In analyzed histological sections, tdTomato red fluorescent signal was detected at all sites (**Figure S2A**), except for the lungs and the brain (**Figure S2B**). The fluorescent signal was particularly enriched in the spleen in which small fluorescent particles were also observed, likely resulting from the parasite trapping and destruction due to the filter activity of this organ (**Figure S2A**).

To assess whether gametocytes had already reached the chimeric ossicle within this short time frame, multiple frozen sections were cut from gelatin-embedded organoids and analyzed by different methods. Transmitted light microscopy on H&E-stained samples revealed stage II-III *P. falciparum* immature gametocytes with pigmented structures consistent with hemozoin (**Figure 2B**). Fluorescent confocal microscopy demonstrated the presence of tdTomato red fluorescent signals in cells that met the morphology (elongated shape) and size (5-10 microns) of stage II-III gametocytes and were characterized by the presence of intracellular dark corpuscles again consistent with hemozoin pigment (**Figure 2C**). Finally, immunofluorescence performed with an antibody recognizing the *P. falciparum* gametocyte-specific protein Pfs16 (Bruce et al., 1994) demonstrated the co-localization of the immunolabeling with the tdTomato red fluorescent signal (**Figure 2D**).

Altogether, these findings clearly showed that stage II-III gametocytes localized in the chimeric ossicles and that this occurred in a few minutes after the injection.

To obtain indirect evidence of the viability of the *P. falciparum* gametocytes that reached the ossicle, we repeated the experiments using *P. falciparum* gametocytes Pf2004/p164-tdTom previously killed by treatment with 2 µM methylene blue (MB) for 24 hours (Adjalley et al., 2011; Siciliano et al., 2017), a treatment that does not abolish the tdTom fluorescent signal from the dead parasites (data not shown). At 10 minutes after injection of MB treated gametocytes, light microscopy analysis of Giemsa-stained blood smears failed to detect parasites in circulation (<0.1 per microliter) and confocal microscopy analysis of ossicles harvested at 10 minutes and 1hr post-injection respectively failed to detect the presence of gametocytes (data not shown). These experiments suggest that the fluorescent parasites found in the ossicles at 10 minutes and later time points after injection were vital stage II-III *P. falciparum* gametocytes.

### *P. falciparum* gametocytes remain in the ossicle and relocate in the extravascular space

**Figure 3:**
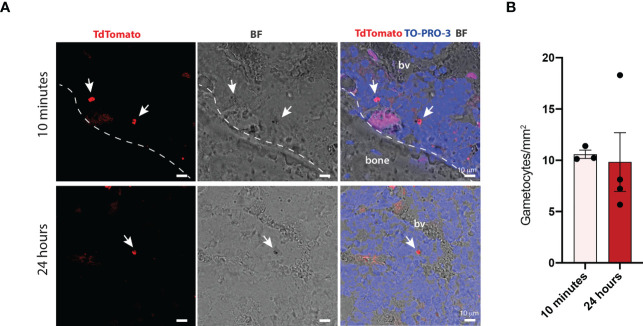
Quantification of *P. falciparum* stage II-III gametocytes within the bone marrow of chimeric ossicles **(A)** Confocal fluorescent images showing stage II-III gametocytes in the bone marrow of ossicles harvested at 10 minutes and 24 hours post-injection (white arrows). White dotted line represents the boundary between the bone and the bone marrow. **(B)** Quantification of stage II-III gametocytes within bone marrow ossicles at the two different time points, each dot represents one ossicle. Ossicles derived from n=2 mice at 10 minutes and from n=2 mice at 24 hours. Data are presented as the mean ± SD. bv, blood vessel.

To investigate whether stage II-III gametocytes could be detected within the ossicle at later time points, confocal microscopy was performed on ossicles harvested at 24 hours. Qualitative (**Figure 3A**) and quantitative (**Figure 3B**) analyses demonstrated that the number of gametocytes at this time was comparable to that observed at 10 minutes, thus indicating that gametocytes that reached the ossicle remained at this site at least for 24 hours.

**Figure 4:**
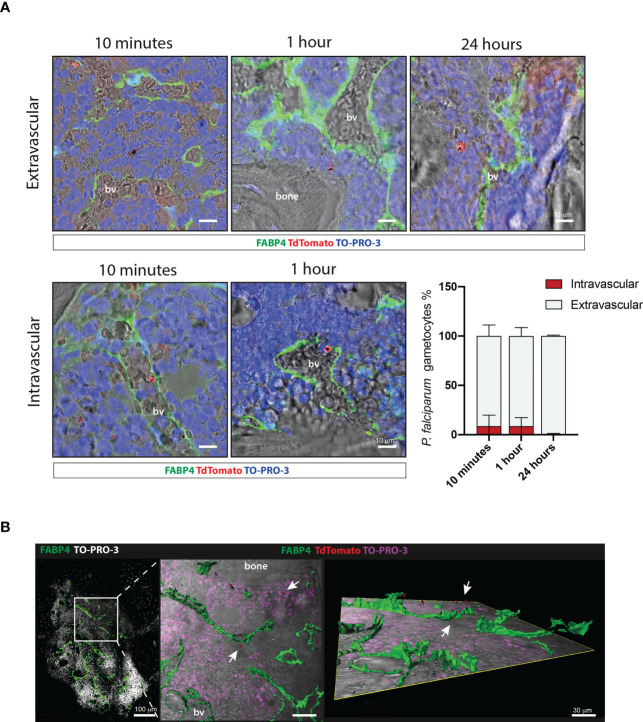
Extravascular and intravascular distribution of *P. falciparum* stage II-III gametocytes within the chimeric ossicle **(A)** Representative confocal images of TdTomato positive stage II-III gametocytes outside (upper panel) and inside (lower panel) blood vessel lumen at 10 minutes, 1 hour and 24 hours. Endothelial cells were stained with an anti-FABP4 antibody. Results of quantitative analysis are also shown (bottom right panel) A total of 177, 104 and 81 gametocytes were counted on FABP4 stained ossicle sections at 10 minutes (4 ossicles from 3 mice), 1 hour (4 ossicles from 2 mice) and 24 hours (4 ossicles from 2 mice), respectively. **(B)** Three-dimensional reconstruction of 50 μm-thick section stained with FABP4 antibody showing the extravascular distribution of stage II-III gametocytes in the ossicle bone marrow. bv, blood vessel.

To evaluate the distribution of gametocytes within the intra- and extra-vascular compartments of the ossicle, histological sections from organoids collected at 10 and 60 minutes, as early time points, and 24 hours, as a late time point, were stained with an anti-FABP4 antibody to label endothelial cells. This analysis showed that about 90% of the red fluorescent, pigmented stage II-III gametocytes were found in extravascular sites at as early as 10 minutes from their infusion [mean ± SD (91,264 ± 11,138)]. The minority fraction of gametocytes within the blood vessel lumen was similar at 10 and 60 minutes [10 minutes: mean ± SD (8,736 ± 11,138); 60 minutes: mean ± SD (8,745 ± 8,577)] while it was further reduced at 24 hours [mean ± SD (0,446 ± 0,893)] (**Figure 4A**). The distribution of gametocytes within the extravascular compartment was further highlighted by three-dimensional confocal microscopy (**Figure 4B**).

These results show that as early as 10 minutes from injection the majority of stage II-III gametocytes localized in the stromal compartment of the ossicle where they were retained in the subsequent hours.

**Figure 5:**
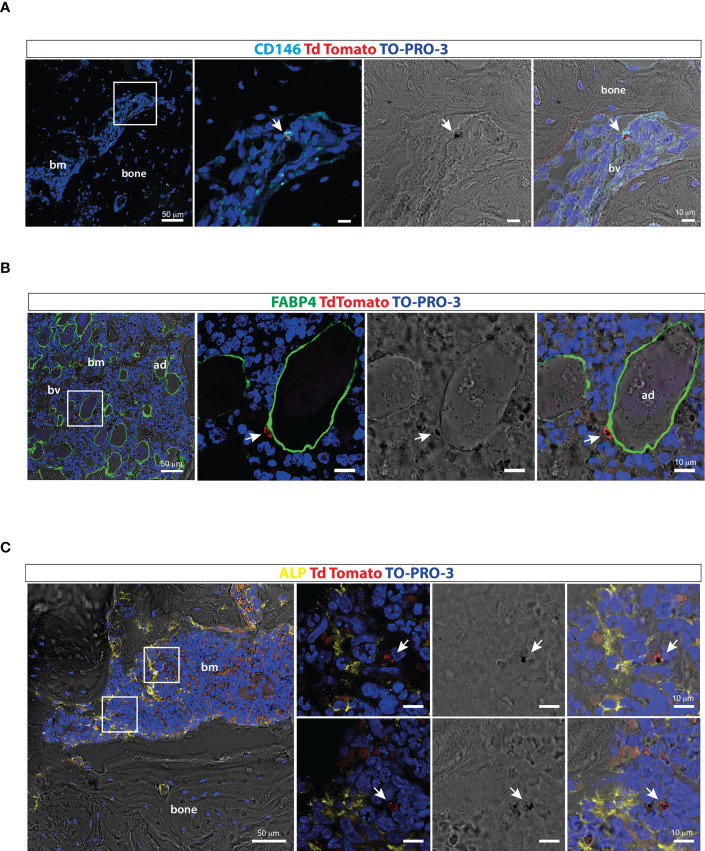
Distribution of *P. falciparum* stage II-III gametocytes within the ossicle bone marrow microenvironment **(A)** Confocal fluorescent images of ossicle sections stained with anti-CD146 showing TdTomato positive stage II-III gametocytes in contact with a human-CD146 positive stromal cell surrounding a murine blood vessel (arrow). **(B)** Confocal fluorescent images of ossicle sections stained with an anti-FABP4 antibody showing a tdTomato immature gametocytes in close contact with a human bone marrow adipocyte (arrow). **(C)** Confocal fluorescent images of ossicle sections stained with an ALP antibody showing TdTomato positive stage II-III gametocytes near ALP-positive osteogenic cells (arrow). bm, bone marrow; bv, blood vessel; ad, adipocyte.

Ossicle sections were immunostained with antibodies recognizing different cellular components of the human BM stroma to preliminarily investigate the distribution of stage II-III gametocytes within the human BM extravascular space. Immunofluorescence performed with an anti-human CD146 and an anti-FABP4 antiserum intriguingly showed that gametocytes can be found closely adjacent to human CD146 positive pericytes (**Figure 5A**) and human FABP4 expressing marrow adipocytes (**Figure 5B**), respectively. A similar observation was made using an anti-ALP antibody, which revealed proximity of gametocytes to human osteogenic stromal cells (**Figure 5C**).

## Discussion

The potential role of the BM niche/microenvironment in supporting virus, bacteria and other agents causing systemic infectious diseases has remained largely unaddressed. Indeed, most studies in the field have been focused on how the BM senses and responds to systemic infections by modulating immune cells and hematopoiesis (Johnson et al., 2020; Gomes et al., 2021). In malaria patients, the BM is not only involved in the response to the inflammatory cascade induced by the infection (Feldman and Egan, 2022), but it is also extensively colonized by the parasite acting as a privileged site for homing and maturation of *P. falciparum* gametocytes (Farfour et al., 2012; Aguilar et al., 2014; Joice et al., 2014).

Localization of gametocytes in the BM is a conserved feature in malaria parasite species, as documented in the murine parasite *P. berghei* (De Niz et al., 2018) and the human parasite *P. vivax* (Obaldia et al., 2018). However, there is a significant evolutionary distance of *P. falciparum* from these species (Liu et al., 2010) as highlighted by the process of blood stage sexual differentiation. Specific features of *P. falciparum* gametocytes, including the long maturation time, the typical falciform morphology and the ability to escape spleen clearance by sequestration, altogether suggest that the *P. falciparum*-host interplay in BM is governed by specific mechanism(s). Indeed, recent studies have unveiled important processes and interactions. For instance, *in vitro* observations have suggested that gametocyte may form directly in the BM, as human erythroblasts can be infected by *P. falciparum* sexually committed parasites and can support gametocyte development (Neveu et al., 2020); other *in vitro* studies have suggested that *P. falciparum* gametocytes, once in the BM stroma, could regulate cytokine production by human BM mesenchymal cells (BM stromal cells, skeletal stem cells) and stimulate BM-derived endothelial cells to form capillary-like structures (Messina et al., 2018). However, the pathophysiological relevance of these findings needs to be confirmed in the natural infections and this requires a measure of emancipation from the need of patients’ material. The development of experimental models reproducing a humanized BM has multiple facets involving the erythropoiesis, the vascular endothelium and the stromal microenvironment, for each of which suitable experimental approaches need to be identified. Currently, the only available study addressing *P. falciparum* gametocyte sequestration in an *in vivo* model has been performed by Duffier et al. (Duffier et al., 2016). These authors have analyzed the distribution of *P. falciparum* gametocytes in the organs of chlodronate-treated NSG immunodeficient mice in which human erythrocytes were infused to support *P. falciparum* sexual development, observing an enrichment of immature gametocytes in the mouse bone marrow and spleen.

The ectopic ossicle generated in immunocompromised mice is a miniature version of bone as an organ in which the skeletal compartments (bone and BM stroma) are of human origin whereas the hematopoietic tissue is provided by the host. The architecture of the human BM is fully reproduced in this chimeric organoid as a result of a timed developmental process in which the stroma and its vascular networks develop prior to colonization by mouse hematopoietic stem/progenitor cells (Sacchetti et al., 2007; Serafini et al., 2014). Previous studies have shown that the ectopic ossicle is efficiently engrafted by human hematopoietic progenitor cells (Pievani et al., 2017) and cancer cells (Antonelli et al., 2016) that circulate the mouse blood. We have now provided a functional probing of its capacity to also host blood-borne *P. falciparum* gametocytes. Following injection of immature gametocytes in mice, previously transplanted with chondroid rudiments, we have harvested the ossicles at different time points to assess the homing, retention and distribution of parasites within the human BM niche.

The bulk biodistribution of gametocytes has confirmed the immediate clearance from mouse blood and the pattern of localization in peripheral organs previously reported in immune-modulated mice (Duffier et al., 2016). *P. falciparum* gametocytes have been detected in the ectopic ossicle at an early time point after the injection with a specific enrichment in the extravascular compartment. Previous studies on bone biopsies from post-mortem specimens and from one clinical case of *P. falciparum* malaria have reported that the majority of stage II-IV gametocytes are found in the BM extravascular space (Farfour et al., 2012; Joice et al., 2014). Our data demonstrate that vital stage II-III *P. falciparum* gametocytes localize in the ossicle and rapidly colonize the extravascular compartment. As the endothelium of the ossicle compartment is of murine origin and as immature (stage I-IV) gametocytes fail to adhere to human endothelial cells (Silvestrini et al., 2012; Tibúrcio et al., 2013), the rapid homing of *P. falciparum* gametocytes in the ossicle strongly indicates, for the first time in an *in vivo* model, that the first steps of gametocyte sequestration in BM are not driven by specific interactions with the host endothelium. In contrast, the ability to rapidly reach the ossicle extravascular compartment draws attention to physical and/or regulatory interactions of *P. falciparum* gametocytes with host cells of the stromal compartment. For instance, the closeness of gametocytes with cells expressing the osteogenic marker ALP provides the first *in situ* evidence, in an *in vivo* model, of the interaction between *P. falciparum* gametocytes and the BM osteogenic stroma, previously reported only in a three-dimensional co-cultures system (Messina et al., 2018). Particularly important was the proximity of gametocytes to human perivascular stromal cells, specifically labelled by the expression of the adhesion molecule CD146, since the pericyte compartment of the human BM stroma is known to include skeletal stem/progenitor cells that are directly involved in the establishment of a niche for normal hematopoiesis and malignant cells (Sacchetti et al., 2007; Antonelli et al., 2016). The localization of immature gametocytes close to BM adipocytes, recognized by morphology and FABP4 expression, confirms the observation made by Farfour et al. on BM biopsies (Farfour et al., 2012). In this context, it is interesting to note that malaria parasites sequestered in peripheral white adipose tissue cause enhanced circulating levels of leptin, an adipokine that has been correlated to the morbidity of cerebral malaria (Mejia et al., 2021). Although BM adipocytes represent a distinct human adipose depot as for development, metabolism and functional properties, Farfour’s and our findings suggest that similar modulatory processes may occur in the adipose marrow. Altogether, our data further reinforce the urgency of suitable experimental models to investigate not only the developmental process of *P. falciparum* gametocytes within the BM but also the effects of the parasite on the non-hematopoietic cell types residing therein to better understand the clinical morbidity of the disease.

In conclusion, we have developed an innovative *in vivo* humanized model to study the host parasite interplay in BM infected by *P. falciparum*. All the fundamental dynamic events associated with BM homing and colonization by the parasite are reproduced and may be investigated in the ectopic ossicles. Furthermore, refinements of the model may be envisioned to address specific questions. For instance, other human components, i.e., erythropoiesis and endothelial cells, may be added to generate a fully humanized system and each component of the system, including relevant parasite genes, may be then manipulated to identify mechanisms of interaction most critical for *P. falciparum* transmission or pathogenesis. Finally, our ossicle-based model, used here for the first time in the field of infectious diseases, represents a powerful tool to study BM function, pathology and host-pathogen interplay in other microbial infections.

## Data availability statement

The raw data supporting the conclusions of this article will be made available by the authors, without undue reservation.

## Ethics statement

The animal study was reviewed and approved by the National Centre for Animal Experimentation and Welfare (BENA) of Istituto Superiore di Sanità and authorised by the Italian Ministry of Health n.574/2017-PR, 17-07-2017.

## Author contributions

Conceptualization: AC, MR, and PA; methodology: MR and PA; investigation: SD, GS, MC, BP, VM, VdT, GF, and FS; resources: SD, GS, MS, AC, MR, and PA; writing – original draft: SD, GS, MR, and PA; writing – review and editing: SD, GS, BP, MS, FS, AC, MR, and PA; funding acquisition: AC, MR, and PA; visualization: SD, GS, BP, VdT, FS, AC, MR, and PA; supervision: MR and PA. All authors contributed to the article and approved the submitted version.
