# Supplementary material for: An in vivo humanized model to study homing and sequestration of Plasmodium falciparum transmission stages in the bone marrow
Source: Front Cell Infect Microbiol. 2023 Apr 19;13:1161669. doi: 10.3389/fcimb.2023.1161669 (PMC10154621; doi:10.3389/fcimb.2023.1161669)
Supplement: Supplementary file 1 [file DataSheet_1.pdf]

Supplementary Material

An in vivo humanized model to study homing and sequestration of *Plasmodium falciparum* transmission stages in the bone marrow

Samantha Donsante[1§], Giulia Siciliano[2§], Mariagrazia Ciardo[2], Biagio Palmisano[1], Valeria Messina[2], Valeria de Turris[3] Giorgia Farinacci[1], Marta Serafini[4], Francesco Silvestrini[2], Alessandro Corsi[1], Mara Riminucci[1^*], Pietro Alano[2^*].

[1] Department of Molecular Medicine, Sapienza University of Rome, Rome, Viale Regina Elena 291, 00161, Italy

[2] Dipartimento Malattie Infettive, Istituto Superiore di Sanità, Rome, Viale Regina Elena 299, 00161, Italy

[3] Center for Life Nano- and Neuro-science Istituto Italiano di Tecnologia, Rome, Viale Regina Elena 291, 00161 Italy

[4] Centro Ricerca M. Tettamanti, Department of Pediatrics, University of Milano-Bicocca, Monza, Via G. B. Pergolesi 33, 20900 Italy.

Supplementary Figures

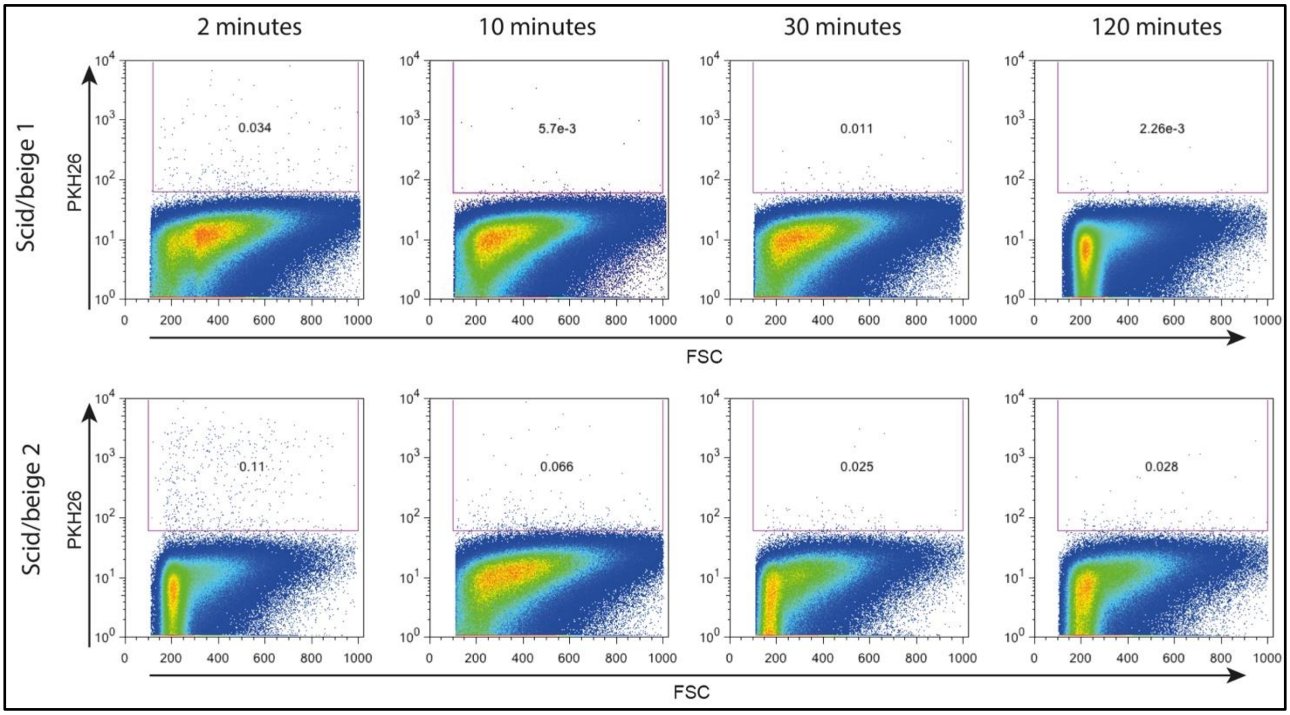

Supplementary Figure S1 _Clearance of PHK26+_ Plasmodium falciparum _stage II-III gametocytes from mouse peripheral blood at 2, 10, 30 and 120 minutes._ Flow cytometry analysis of blood collected from 2 SCID/beige mice after 2, 10, 30, and 120 minutes after PHK26+ stage II-III gametocytes injection.

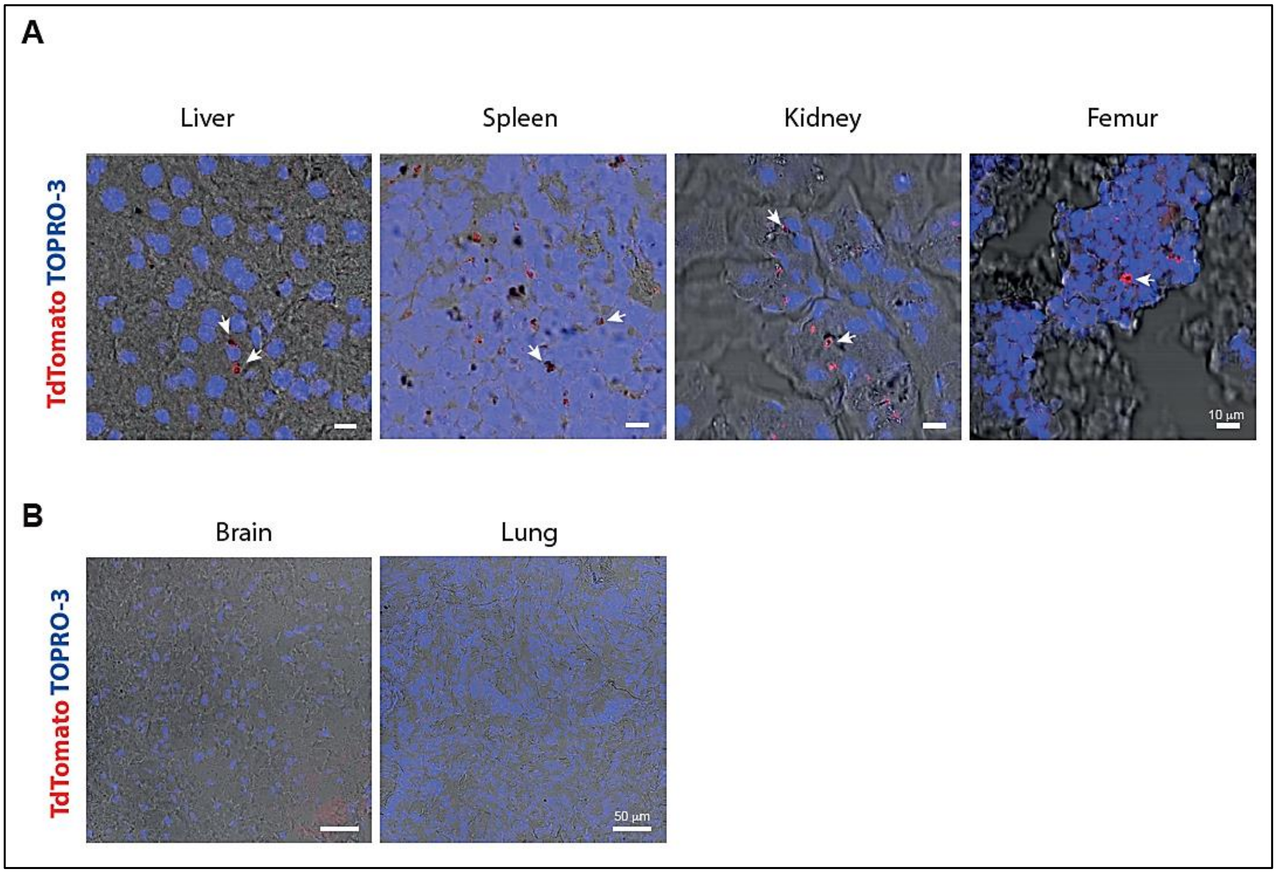

Supplementary Figure S2 *Distribution of* Plasmodium falciparum *stage II-III gametocytes within mouse organs at 10 minutes post-injection.* A) Representative confocal images of TdTomato Stage II-III gametocytes in liver, spleen, kidney and bone marrow (white arrows) from 2 mice. B) Representative images of murine brain and lung showing no TdTomato signal or pigmented structures.
